# Magnetic resonance neurography–supported L3–L4 radiculitis presenting as refractory peripatellar neuropathic pain in a patient with diabetes: A case report

**DOI:** 10.1097/MD.0000000000051406

**Published:** 2026-11-06

**Authors:** Xue Huang, Dan Yang, Jie Wang, Bixin Zheng

**Affiliations:** a Department of Pain Management, West China Hospital, Sichuan University, Chengdu, Sichuan Province, China; b Department of Pain Management, West China Tianfu Hospital, Sichuan University, Chengdu, Sichuan Province, China.

**Keywords:** case report, diabetes mellitus, L3–L4 radiculitis, magnetic resonance neurography, neuropathic pain, peripatellar pain

## Abstract

**Rationale:**

Peripatellar pain is usually attributed to local knee disorders. However, proximal neural lesions can also cause pain around the knee. Third and fourth lumbar (L3–L4) radiculitis presenting mainly as peripatellar neuropathic pain is uncommon and may be missed when typical radicular symptoms are absent.

**Patient concerns:**

A 75-year-old man with a more than 20-year history of type 2 diabetes presented with severe left peripatellar pain for 10 days. The pain was burning and stabbing and was accompanied by numbness and marked touch-evoked allodynia. The visual analog scale score was 8–9/10, and the Douleur Neuropathique 4 score was 9/10. He had no low back pain, typical radicular pain, motor weakness, or reflex abnormalities.

**Diagnoses:**

Knee magnetic resonance imaging showed degenerative changes that did not explain the neuropathic pain phenotype. Lumbar magnetic resonance imaging showed no definite compression of the L3 or L4 nerve roots. Electrodiagnostic testing did not provide level-specific localization. Magnetic resonance neurography showed asymmetric hyperintensity involving the left L3 and L4 proximal neural structures, supporting the diagnosis of L3–L4 radiculitis.

**Interventions:**

Neuropathic pain medications and knee-targeted procedures provided inadequate or only transient relief. The patient was subsequently treated with pregabalin, duloxetine, short-term opioid analgesia, and optimization of glycemic control.

**Outcomes:**

The pain and allodynia gradually improved within 2 weeks, and analgesic medications were discontinued. At 3 months, the visual analog scale score was 0–3/10 and the Douleur Neuropathique 4 score was 1/10. Touch-evoked allodynia had resolved.

**Lessons:**

L3–L4 radiculitis may present as refractory peripatellar neuropathic pain without low back pain or typical radicular symptoms. When severe neuropathic pain around the knee cannot be explained by local pathology, a proximal neural lesion should be considered. Magnetic resonance neurography may help identify radiculitis that is not evident on conventional lumbar imaging.

## 1. Introduction

Peripatellar pain often prompts evaluation for local knee disorders, such as osteoarthritis, meniscal degeneration, or periarticular soft-tissue lesions. However, structural abnormalities on knee imaging do not always correspond to the location or severity of pain.^[1–3]^ Neural lesions should, therefore, be considered when the clinical phenotype cannot be adequately explained by local pathology. The sensory innervation of the knee is complex and involves multiple neural pathways.^[4]^ The third and fourth lumbar nerve roots (L3 and L4) contribute to sensory innervation of the anterior thigh and medial knee region, and L3 radiculopathy can present with knee pain.^[4,5]^ Neuropathic features, including burning pain, numbness, hyperalgesia, and allodynia, should further raise suspicion of a neural origin. Lumbar radicular disease commonly presents with radicular pain, sensory disturbance, motor deficits, or a combination of these findings.^[5,6]^ An isolated peripatellar presentation is uncommon and may lead to an initial focus on local knee pathology.

Diabetes mellitus is associated with a wide spectrum of peripheral neuropathies. Distal symmetric polyneuropathy is the most common form, but focal and proximal neuropathies involving nerve roots, plexuses, and individual peripheral nerves can also occur.^[7–9]^ Metabolic, microvascular, and inflammatory mechanisms have been implicated in these neuropathic syndromes.^[8–10]^ Unlike compressive radiculopathy, inflammatory or metabolic neural lesions may not be apparent on conventional lumbar magnetic resonance imaging (MRI). Magnetic resonance neurography (MRN) allows direct visualization of neural structures and can demonstrate abnormalities in nerve signal and morphology. It may, therefore, provide additional diagnostic information when routine imaging and electrodiagnostic testing are inconclusive.^[11–13]^

We report a patient with long-standing diabetes who developed severe refractory neuropathic pain confined to the peripatellar region. He had no low back pain, typical radicular radiation, motor weakness, or reflex abnormalities. Knee MRI showed degenerative changes that did not explain the neuropathic pain phenotype, and conventional lumbar MRI showed no definite L3 or L4 nerve-root compression. MRN subsequently demonstrated abnormal signal involving the left L3–L4 neural structures, supporting the diagnosis of L3–L4 radiculitis. This case highlights an unusual presentation of lumbar radiculitis and illustrates the potential role of MRN in identifying an otherwise occult cause of refractory peripatellar neuropathic pain.

## 2. Case presentation

### 2.1. Patient information and clinical findings

A 75-year-old man was admitted with severe pain around the left patella that had persisted for 10 days. He had a more than 20-year history of type 2 diabetes mellitus and was receiving insulin therapy. Glycemic control was suboptimal, with a glycated hemoglobin level of 8.6%. Before admission, tramadol and diclofenac had provided little pain relief.

The pain was localized mainly to the medial and lateral peripatellar regions. It was continuous, burning, and stabbing and was accompanied by numbness and marked touch-evoked allodynia. Even light contact with clothing aggravated the pain. The visual analog scale (VAS) score was 8–9/10, and the Douleur Neuropathique 4 score was 9/10. The pain was not clearly related to knee movement. The patient denied low back pain or typical radiating pain involving the anterior or medial thigh or the medial lower leg.

Physical examination revealed marked tactile allodynia over the anterior peripatellar region. There was no redness, swelling, or limitation of knee movement. Neurological examination showed no definite motor weakness, dermatomal sensory loss, or reflex abnormality. No vasomotor, sudomotor, trophic, or motor changes suggestive of complex regional pain syndrome were observed. No vesicular eruption was present. Routine laboratory investigations showed no evidence of infection, gout, or systemic inflammation.

### 2.2. Initial diagnostic assessment and treatment

MRI of the left knee showed osteoarthritic changes, focal grade IV chondral defects involving the lateral tibial plateau and patella, grade II meniscal degeneration, mild joint effusion, and swelling of the suprapatellar fat pad (Fig. S1, Supplemental Digital Content 1). However, these structural abnormalities did not adequately explain the acute onset and prominent neuropathic features of the pain. Electrodiagnostic testing showed sensory-predominant peripheral neurogenic abnormalities but did not provide level-specific localization (Table S1, Supplemental Digital Content 2).

Initial treatment included pregabalin, opioid analgesics, and topical lidocaine. Pain control remained inadequate. Given the peripatellar distribution of the pain, ultrasound-guided genicular nerve blocks were performed at the superomedial, inferomedial, superolateral, and inferolateral genicular nerve regions. At each target, 2 mL of a local anesthetic mixture containing 0.25% lidocaine and 0.3% ropivacaine was injected. The VAS score decreased from 8–9/10 to 2–3/10, but the analgesic effect lasted for only approximately 8 hours. Because of the marked but short-lived response to the nerve block, pulsed radiofrequency was subsequently performed at the same 4 targets at 42°C and 100 volts (V) for 5 minutes per target. Pain relief lasted for approximately 1 day. An intra-articular injection containing 2 mL of 0.5% lidocaine and 2.5 mL of sodium hyaluronate (25 mg) was also administered. No clinically meaningful early pain relief was observed. The persistent neuropathic pain and limited response to knee-targeted treatment prompted further investigation for a proximal neural cause.

### 2.3. MR neurography and diagnosis

Lumbar MRI showed L3–L4 disc bulging with protrusion and L4–L5 disc bulging. However, there was no definite compression of the L3 or L4 nerve roots (Fig. S2, Supplemental Digital Content 3). These findings did not adequately explain the severity or distribution of the patient’s neuropathic pain.

Lumbosacral plexus MRN was subsequently performed using a 3.0-T MRI system. MRN demonstrated asymmetric hyperintensity of the left L3 and L4 anterior rami compared with the contralateral side (Fig. 1). In the context of the marked neuropathic pain phenotype, absence of definite nerve-root compression, and long-standing diabetes, the MRN abnormalities were considered evidence of inflammatory involvement of the left L3–L4 neural pathway. A clinical diagnosis of noncompressive L3–L4 radiculitis was therefore made.

**Figure 1.**
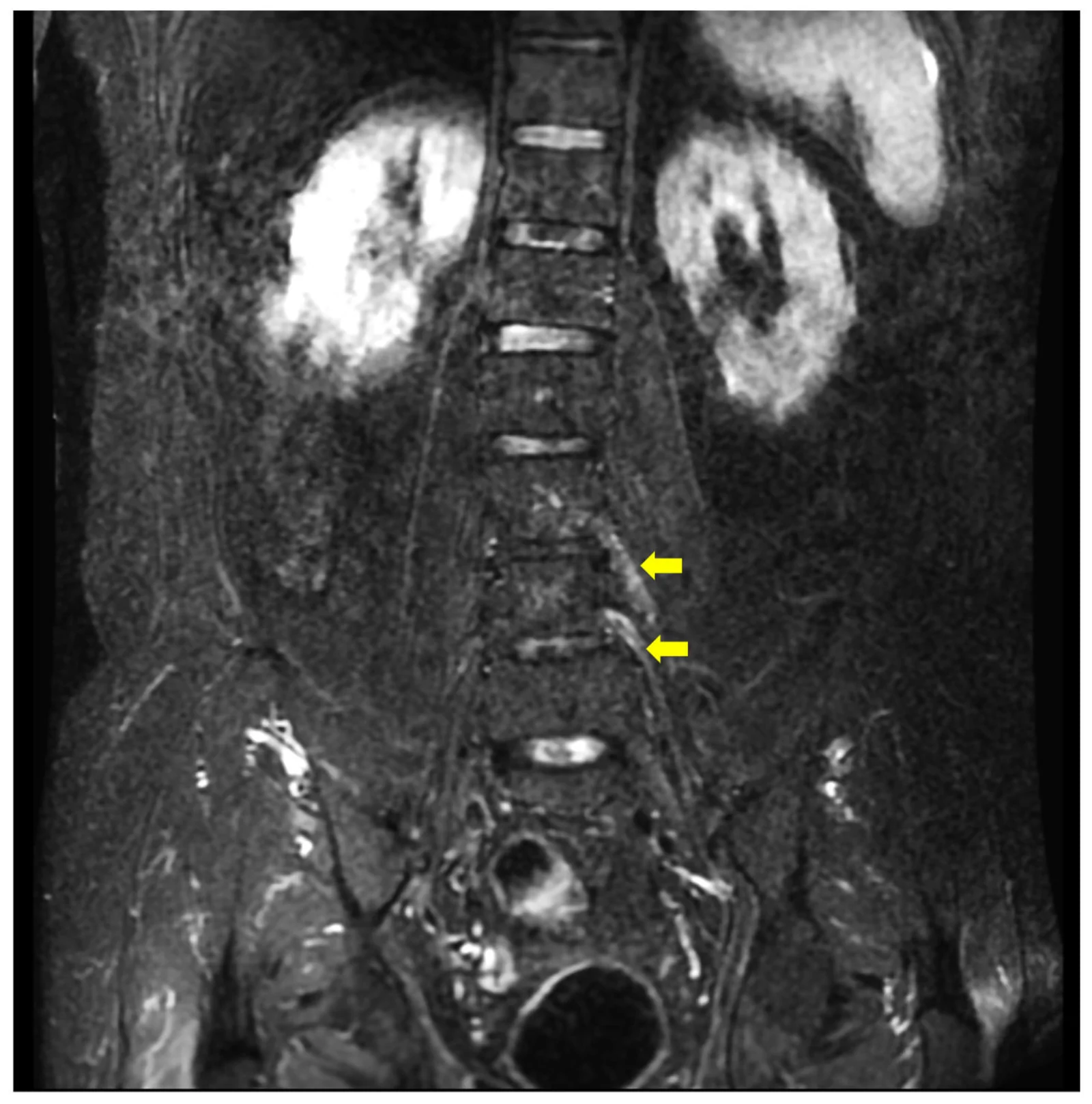
Lumbosacral plexus MRN. Coronal MRN images acquired on a 3.0-T MRI system demonstrate asymmetric hyperintensity of the left L3 and L4 anterior rami compared with the contralateral side. Arrows indicate the hyperintense left L3 and L4 anterior rami. MRI = magnetic resonance imaging, MRN = magnetic resonance neurography.

### 2.4. Treatment and follow-up

Treatment was continued with pregabalin and duloxetine, with short-term opioid analgesia as needed. Glycemic control was also optimized. The pain and allodynia gradually improved over the following 2 weeks, after which analgesic medications were discontinued.

At the 3-month follow-up, the VAS score was 0–3/10 and the Douleur Neuropathique 4 score had decreased to 1/10. Touch-evoked allodynia had resolved. The patient was able to walk, dress, and sleep without substantial pain interference. No vesicular eruption developed during follow-up, and no procedure-related adverse events were observed.

## 3. Discussion

This case describes an unusual presentation of L3–L4 radiculitis in a patient with long-standing diabetes. The predominant symptom was severe neuropathic pain confined to the peripatellar region. Typical features of lumbar radicular disease, including low back pain, proximal radiating pain, motor weakness, and reflex abnormalities, were absent. Knee MRI showed degenerative changes, but these findings did not adequately explain the acute neuropathic phenotype. Structural abnormalities on knee imaging do not always correlate closely with the location or severity of pain.^[2,3]^ Conventional lumbar MRI also showed no definite nerve-root compression. MRN subsequently demonstrated abnormalities involving the left L3–L4 neural structures and provided an anatomical correlate for the otherwise unexplained pain.

The relationship between L3–L4 neural involvement and peripatellar pain is anatomically plausible. The L3 and L4 neural pathways contribute to the femoral and saphenous nerve systems and participate in sensory innervation of the anterior and medial aspects of the knee.^[4,5]^ The sensory innervation of the anterior knee is complex and involves several overlapping articular and cutaneous branches.^[4,14]^ A proximal neural lesion may, therefore, not produce a classic dermatomal pattern. Previous clinical observations have shown that L3 radiculopathy can present predominantly as hip or knee pain and may initially be mistaken for local joint disease.^[5]^ In the present patient, pain was largely confined to the peripatellar region and was not accompanied by typical thigh or lower-leg radiation. This atypical distribution likely contributed to the initial focus on local knee pathology.

Radiculitis should also be distinguished from compressive radiculopathy. Lumbar radiculopathy is commonly associated with structural abnormalities, particularly disc herniation and foraminal narrowing, that irritate or compress the affected nerve root.^[15]^ In contrast, inflammatory or metabolic neural injury may occur without obvious mechanical compression. This distinction may be particularly relevant in patients with diabetes. Diabetes is associated with several forms of focal and proximal neuropathy in addition to distal symmetric polyneuropathy.^[7–9]^ Microvascular ischemia, metabolic injury, and inflammatory mechanisms have all been implicated in diabetic neuropathic syndromes.^[8–10]^ In our patient, long-standing diabetes and suboptimal glycemic control may have increased susceptibility to proximal neural injury. However, a direct causal relationship between diabetes and L3–L4 radiculitis cannot be established from a single case.

MRN played an important role in identifying the proximal neural abnormality. Conventional lumbar MRI is primarily used to assess vertebral, disc, spinal canal, and foraminal pathology. It may, therefore, provide limited information when symptoms arise from noncompressive neural disease. MRN is optimized for direct visualization of peripheral nerves and plexuses and can provide information about nerve morphology and signal characteristics.^[11–13,16]^ Increased T2-weighted signal, nerve enlargement, and asymmetry are recognized MRN findings in peripheral nerve disorders.^[11,16]^ Quantitative MRN studies have also demonstrated increased signal intensity and enlargement of lumbosacral nerve roots in inflammatory polyradiculoneuropathies.^[17]^ In the present case, MRN showed asymmetric hyperintensity involving the left L3 and L4 anterior rami despite the absence of definite nerve-root compression on conventional lumbar MRI. These findings, together with the neuropathic pain phenotype and clinical course, supported the clinical diagnosis of L3–L4 radiculitis. Nevertheless, increased neural signal on MRN is not disease-specific and should be interpreted in conjunction with clinical and electrophysiological findings.

Several alternative diagnoses were considered. Degenerative knee disease was unlikely to be the main pain generator because the clinical presentation was dominated by burning pain, numbness, and marked allodynia rather than movement-related mechanical pain.^[2,3]^ A distal sensory neuropathy involving the saphenous nerve or its infrapatellar branch can also produce neuropathic pain around the knee.^[18,19]^ However, there was no preceding trauma, surgery, or clear evidence of focal entrapment. Diabetic lumbosacral radiculoplexus neuropathy was another important consideration. This disorder typically begins with severe unilateral pain in the hip or thigh and is often followed by progressive weakness and muscle atrophy.^[8,9]^ These features were absent in our patient. Complex regional pain syndrome was considered unlikely because the patient had no vasomotor, sudomotor, trophic, or motor abnormalities characteristic of the disorder.^[20]^ Herpes zoster–related neuralgia, including zoster sine herpete, was also considered. Zoster sine herpete can occur without a vesicular eruption.^[21]^ In this patient, no eruption developed during follow-up, and there were no additional clinical findings to support varicella-zoster virus reactivation. Virological testing was not performed.

This case has several limitations. First, no selective L3 or L4 nerve-root block was performed to provide additional functional confirmation of the involved level. Second, electrodiagnostic testing did not provide level-specific localization. Third, MRN abnormalities were evaluated qualitatively, and follow-up MRN was not performed after clinical improvement. In addition, pain relief may have resulted from the combined effects of neuropathic pain medications, previous interventional procedures, improved glycemic control, and the natural course of the disease. Finally, the association between diabetes and L3–L4 radiculitis remains inferential. Despite these limitations, this case illustrates an important diagnostic consideration: severe peripatellar neuropathic pain may arise from proximal L3–L4 neural disease even when typical radicular symptoms and compressive findings are absent.

## 4. Conclusion

Refractory peripatellar neuropathic pain may rarely be the presenting manifestation of L3–L4 radiculitis. This diagnosis can be overlooked when low back pain, typical radicular symptoms, and compressive abnormalities on conventional lumbar MRI are absent. In patients with diabetes and unexplained neuropathic pain around the knee, MR neurography may reveal an occult proximal neural lesion and help establish the underlying diagnosis.

## Acknowledgments

This work was supported by the 1·3·5 Project for Disciplines of Excellence–Clinical Research Fund, West China Hospital, Sichuan University (grant number: 2023HXFH035).

## Author contributions

**Data curation:** Xue Huang, Jie Wang, Bixin Zheng.

**Validation:** Xue Huang, Bixin Zheng.

**Visualization:** Xue Huang.

**Conceptualization:** Dan Yang, Bixin Zheng.

**Supervision:** Bixin Zheng.

**Writing – original draft:** Xue Huang, Dan Yang, Bixin Zheng.

**Writing – review & editing:** Dan Yang, Jie Wang, Bixin Zheng.






