# Supplementary material for: Magnetic resonance neurography–supported L3–L4 radiculitis presenting as refractory peripatellar neuropathic pain in a patient with diabetes: A case report
Source: Medicine (Baltimore). 2026 Nov 6;105(45):e51406. doi: 10.1097/MD.0000000000051406 (PMC13747597; doi:10.1097/MD.0000000000051406)
Supplement: Supplementary file 1 [file medi-105-e51406-s002.docx]

Table S1. Electrodiagnostic findings.

| **Nerve** | **Side** | **Segment** | **Latency (ms)** | **SNAP amplitude (μV)** | **Conduction velocity (m/s)** |
| --- | --- | --- | --- | --- | --- |
| Superficial peroneal | Left | Dorsum of foot–calf | 2.01 | 9.1 | **42.3** |
| Superficial peroneal | Right | Dorsum of foot–calf | 1.73 | 10.7 | **43.4** |
| Sural | Left | Lateral malleolus–calf | 2.64 | 9.9 | **39.8** |
| Sural | Right | Lateral malleolus–calf | 2.36 | 9.3 | **40.3** |

Abbreviation: SNAP, sensory nerve action potential.
